# Licensing the future: report on BioMed Central’s public consultation on open data in peer-reviewed journals

**DOI:** 10.1186/1756-0500-6-318

**Published:** 2013-08-21

**Authors:** Iain Hrynaszkiewicz, Stefan Busch, Matthew J Cockerill

**Affiliations:** 1 Faculty of 1000, London, UK; 2 BioMed Central Ltd, 236 Gray’s Inn Road, London WC1X 8HB, UK

## Abstract

We report the outcomes of BioMed Central’s public consultation on implementing open data-compliant licensing in peer-reviewed open access journals. Respondents (42) to the 2012 consultation were six to one in favor (29 in support; 5 against; 8 abstentions) of changing our authors’ default open access copyright license agreement, to introduce the Creative Commons CC0 public domain waiver for data published in BioMed Central’s journals. We summarize the different questions we received in response to the consultation and our responses to them – matters such as citation, plagiarism, patient privacy, and commercial use were raised. In light of the support for open data in our journals we outline our plans to implement, in September 2013, a combined Creative Commons Attribution license for published articles (papers) and Creative Commons CC0 waiver for published data.

## Introduction

Respondents to BioMed Central’s public consultation on Open Data [1], which was open for comments from September to November 2012, were six to one in favor of adopting a new copyright system in journal publishing: a system which should increase the efficiency of knowledge discovery from the published literature, which requires little change in scientists’ current behaviors to implement while affecting nearly every article we publish in the future, and which could be adopted by other publishers for the benefit of science. Since the consultation we have been investigating the different technical and procedural approaches for implementing a new license agreement for all BioMed Central journals, in collaboration with our previously established Publishing Open Data Working Group [2].

We are excited by the support for the proposals but must also address any questions that are raised by our authors and editors. Further below we summarize the different questions we received and our responses to them.

Central to the proposals, which we distributed for public consultation, were to change BioMed Central’s standard copyright license agreement for open access articles so that any data in published articles and in additional files are published under the Creative Commons CC0 waiver, rather than the Creative Commons Attribution license, CC-BY (for full details see [3]). Creative Commons CC0 is a public domain dedication and means that a person has dedicated a work “to the public domain by waiving all of his or her rights to the work worldwide under copyright law, including all related and neighboring rights, to the extent allowed by law” [4].

## Summary of responses to the consultation

See Table 1 for a summary of the responses and the primary stakeholder group of each respondent. We did not seek consent for the responses received by email to be published but have collated them and identified the common questions which emerged in an anonymous fashion. A number of these questions were anticipated and discussed in our article in *BMC Research Notes*[3]. And while the questions were mostly valid with regard to sharing and publication of data in general, the majority did not apply in the context of the consultation, which was purely about licensing of data already planned to be made available freely online. We therefore need to make it abundantly clear to authors that the proposed change to our standard license agreement only affects data which authors are already publishing open access, under a Creative Commons Attribution license (our standard license).

**Table 1:** Number of responses received to the consultation, the primary stakeholder group of the respondents and the number who supported, were against or were neutral about the proposals

**Stakeholder group**	**Number of respondents**
Journal editor	23
Author	15
Funder	2
Librarian	2
**Totals**	**42**
Support proposed change	29
Do not support proposed change	5
Abstained	8

The questions fell largely into the following areas.

**Question**: *Will commercial organizations benefit from use of public domain data?*

**Response**: It is already possible for commercial organizations to use content published in open access journals under the CC-BY license for their own benefit. BioMed Central, and many other open access publishers, use CC-BY as the default license for journal articles and their supplementary material (additional files, which can include data). The Open Access Scholarly Publishers Association (OASPA) strongly recommends use of CC-BY [5] by all its members. Using CC0 for data contained in published articles does not change the already existing potential for commercial uses of the published work.

Moreover, permitting commercial use of open access content enables all reuses, including sharing of content on Wikipedia (which uses CC-BY ShareAlike 3.0) and preservation of content by commercial organizations, which could prove valuable [6] in the event of a publisher going out of business. The UK Government has recognized the benefits to the wider economy and, ultimately, tax payers by making publicly-funded data available openly to stimulate business innovation in funding the start-up of the Open Data Institute [7], which launched in 2012.

Applying CC0 to data published in journals is not intended to change the numerous community or journal data availability policies [8]. Authors and editors remain in control of what data they choose to publish, unless they are subject to a community-specific requirement for data release.

**Question**: *Will plagiarism increase?*

**Response**: Plagiarism (unattributed copying) and the potential for plagiarism has increased with digital access to content [9], independent of content licenses. In scholarly publishing plagiarism usually occurs when text, rather than data, is reused without permission or attribution. Under the proposals the license, CC-BY, under which narrative text of articles is published will not change. If data published in journals are available under CC0, re-users of the data should still cite their sources whenever it is technically possible to do so. Software, such as CrossCheck, exists to detect plagiarism, and peer reviewers can also detect plagiarism. Both peer review and plagiarism detection software are agnostic to content licenses. The Creative Commons have rightly described plagiarism as “a completely orthogonal issue to copyright infringement” [10].

**Question**: *Do authors need to publish more data than they publish already?*

**Response**: We are not requiring authors to publish more of their data. The change in license only affects data that authors choose to submit to our journals for open access publication, and does not require release of any other data or a change in license of any data not submitted to the journal. Therefore, authors, editors and their communities remain in control of what content they publish. CC0 is the default term for data which are already being or will be made available open access. However, BioMed Central supports data sharing and release from all areas of research, where this is possible.

**Question**: *What if authors are not allowed, by their funders or employers, to use CC0 for any of their published work?*

**Response**: Where legitimate reasons exist for authors to be unable to apply CC0 to their published data, it is possible to opt out and use a non-standard license. This process already happens in journal publishing. Commonly figures, tables or charts are reproduced, with permission, in journal articles from sources which are licensed differently to the secondary publisher’s terms – and statements to this effect included in articles. When submitting work to journals authors already have read the publisher’s standard copyright and license agreement and, if they cannot agree to the terms, query these before submission or publication. Some scientists funded by the World Health Organization, UK Government, and US Government already have agreements with publishers to use a non-standard copyright statement in their open access articles.

**Question**: *Will patient privacy be put at risk*?

**Response**: Protecting human subjects’ right to privacy is a core principle of ethical research, and of the laws of many countries. The introduction of CC0 does not affect processes and laws relating to informed consent, privacy, and consent for publication. Changing the licensing of freely available data neither affects what human subjects data are submitted for publication nor the accessibility of any anonymized human data which are published [11].

**Question**: *Will articles receive fewer citations?*

**Response**: Applying the CC0 waiver to published data means that legally there is no requirement for attribution of the original author(s) if the data are copied, redistributed or reused. However, anyone reusing data should, whenever technically feasible, still cite the original author(s). Attribution is a legal requirement of copyright law and citation is a cultural norm in scholarship which ensures scientists receive credit for their work. But the two concepts are different and often confused. Citing sources is an established cultural norm in scholarship which has persisted for centuries in the absence of legal requirements for citation. Attribution and citation can sometimes be achieved in the same manner but the practices serve different purposes (see the table in Hrynaszkiewicz & Cockerill [3] for practical examples). Attribution does not always equal citation, and credit in scholarship is assigned by the latter.

Placing data or any other content in the public domain is not incompatible with the generators of the data requesting – non-legal – conditions for its reuse. For example, the International Stroke Trial investigators, who published a large clinical trial dataset under CC-BY, additionally requested “*any publications arising from the use of this dataset acknowledges the source of the dataset, its funding and the collaborative group that collected the data*” [12]. Two other research groups have since reused the data [13].

We are not aware of empirical evidence that applying CC0 to published data results in scientists receiving fewer citations to or less credit for their articles. In fact, the limited evidence available on citation share for published articles which provide full access to supporting data compared to articles with no supporting data suggests that publishing data with journal articles and enabling reuse increases the number of citations. This has been found in microarray research [14], astronomy and the marine sciences [15], although these studies did not evaluate different content licenses – only accessibility.

Furthermore, the attribution requirement is only waived for published *data*, which includes data in additional files and within journal articles. The remainder of each article will retain a CC-BY license.

**Question**: *What incentive is there for the original author(s) to use CC0 instead of CC-BY?*

**Response**: The impact of different licenses for data on citation of datasets and related scholarly works has not yet been established. However, since public domain dedication maximizes the potential for data discovery and reuse we might reasonably hypothesize that open licensing might *increase* individual credit and citations. There is evidence [14-16] that sharing of research data underlying journal articles increases citation share and increases reproducibility of results [17]. A lack of datasets which can be readily shared and combined – *i.e.*, are in the public domain under an open data license – has been identified as hampering progress in evolutionary magnetic resonance imaging (evoMRI) research [18]. Data supporting publications and placed in the public domain in fields facing this problem promote collaboration between research teams and furthers progress.

**Question**: *Why do we need to change the license if copyright already does not apply to data?*

**Response**: We are part of a global research and open access publishing enterprise and whether copyright applies to data varies depending on the legal jurisdiction. In the US this concern may be valid as copyright does not apply to facts (and data are numerical representations of facts), only to the way in which they are presented. However, in Australia copyright could apply to data [19] as the focus of the law is on originality rather than creativity. Furthermore, public domain dedication is not just about copyright. Applying CC0 aims at removing all legal barriers to sharing and reuse of content, and so waiving not just copyright but also all related and neighboring rights, such as patents and trademarks, maximizes the potential for reuse.

Another important reason for implementing explicit and clear open data licensing is about removing ambiguity. For data reuse to be efficient, humans and machines need content to be clearly licensed. The alternative, making case-by-case assessments and checking with individual data publishers and authors about the license or copyright status of individual data packages, does not scale. Being clear about licensing also reduces the risk that an individual or organization publishing or reusing data in good faith does not become involved in unintended legal debate in the future.

**Question**: *Will data storage problems be created for the publisher or authors?*

**Response**: Our open data policy is purely about changing the license for data published in BioMed Central journals. There are no plans to increase the maximum additional file size and number of files which can be published (virtually unlimited files of up 20 Mb per file). Therefore data storage is unaffected by the policy.

## Limitations of the consultation

The consultation ran for two months and was featured on the BMC Blog and BMC Update newsletter. We also contacted the editors of all our independent journals. The response rate to the consultation was therefore fairly low, and as with all surveys and consultations responder bias should be considered. Perhaps we could hypothesize that many scientists read the proposals and understood that what we proposed does not represent a major change (it doesn’t), but this is speculative. We are aiming to provide clarity about the copyright status of content which scientists already choose to make open access and permit all types of reuses, including commercial use. But if at the same time more awareness is raised of the opportunities resulting from more open science, this could be considered an indirect benefit.

## What next

We remain committed to implementing open data compliant licensing in our journals and are now working on the technical and legal details. We defined in our September article the minimum and desirable publishing platform developments that would be needed.

However, one aspect of the public consultation which attracted few responses was the question of “How do you define data?”. This is important as applying a legal tool selectively to different parts of a published work could, in principle, necessitate defining which parts are covered by which tool. But data are notoriously difficult to define. Implementing this at scale in material submitted to journals requires the process to be automated, without the need for humans to evaluate each file type and its contents. Publishers receive a huge variety of file types as supplementary file submissions. There are a number of file types which are more obviously associated with data but comprehensively defining them might be an insurmountable challenge.

Initially, we will therefore simply change our policy so that authors apply CC0 by default to all data included in each article, its reference list(s) and its additional files (including tables, graphical data points, bibliographic data, and machine-harvestable terms), unless an author has opted out. This implementation of an “open by default” license for data makes the approach scalable. So our new standard license statement will read in each article:

“© 2013 < Author > et al. This is an Open Access article distributed under the terms of the Creative Commons Attribution License (http://creativecommons.org/licenses/by/2.0), which permits unrestricted use, distribution, and reproduction in any medium, provided the original work is properly cited. The Creative Commons Public Domain Dedication waiver (http://creativecommons.org/publicdomain/zero/1.0/) applies to the data made available in this article, unless otherwise stated.”

This approach allows re-users of data (humans and machines) to interpret the license in their – in all likelihood good – understanding of data definitions for their area of research. This approach will be complemented by providing even more practical examples of different data types and (re)use cases in our guidelines and Frequently Asked Questions, which will grow over time.

Technology will further enhance the process of attaching licenses to different parts of published articles. Any additional files uploaded on journal submission systems specifically as or tagged as supporting data files would be tagged with CC0 as the default. This human and machine readable licensing information can then attach to and follow each file through to eventual publication and ideally be embedded within the files themselves.

On a related note, we also intend to upgrade the attribution component of BioMed Central‘s license agreement from CC-BY 2.0, which we introduced as our standard license in 2004, to CC-BY 4.0 [20] shortly after the new version will be released following final discussions at the Creative Commons Global Summit which takes place in August 2013. Creative Commons update their licenses every few years in the light of user feedback and technical and legal developments, and CC-BY 4.0 is such an update to the Attribution license.

## Appendix

### Background to the consultation

The focus of our work on promoting data sharing and data reuse has been about removing barriers: making it easier to share science and helping to demonstrate the value of more open approaches to scientific discourse, when these are compatible with community norms, ethical codes and legal statutes. Part of a publisher’s role is to help the scientific community and funders to receive collective community benefit from published science. Open access to journal articles and underlying data, with the use of appropriate open content licenses, should ensure both society and individuals gain the maximum benefit from scientific endeavors. But ‘open’ in open data, and open access, means much more than just access [21].

In 2010 BioMed Central publicly endorsed the Panton Principles for Open Data in Science and issued a draft open data statement which made some initial proposals as to how these principles could be put into practice in journal publishing [22]. At that time, no data published in online journals and their supplements were compliant with these principles. Central to the Panton Principles is ensuring data can be reused, integrated and built upon with the minimum of restrictions. For data which are or which will be free to access online this means dealing with licensing, copyright and intellectual property – and placing data in the public domain by waiving copy and other rights. A widely-accepted tool for doing this is the Creative Commons CC0 waiver. Data repositories such as Dryad and Figshare already use CC0 for data deposits. In our draft open data statement we proposed that in the future all authors could agree that any data which they submit to a journal for open access publication (such as additional data files/supplementary materials, and tables) would be placed in the public domain with a CC0 waiver.

This approach was supported by the consensus of attendees of our Publishing Open Data Working Group meeting, convened in June 2011 after we publicly invited the scientific and publishing community to help us put the Panton Principles into practice. However, the consensus of the working group was also that much more detail needed to be added to the proposal. The case for why authors should do this and the implications of the changes for authors and publishers needed to be made. Therefore, in 2012, with the input of several members of the working group, a detailed paper was published in *BMC Research Notes*[3]. We then invited the public to comment on the proposals, which were extended to explicitly include opening up bibliographic data, and systematically contacted editors of our journals requesting their and their communities’ views. We received comments directly on the blog announcement although the majority were received by email.

## Competing interests

MJC and SB are employed by BioMed Central; IH was employed by BioMed Central at the time of writing this article and is now employed by Faculty of 1000, where CC0 is already in use for their journal F1000Research.
